# The Importance of Larval Stages for Considering Crab Microbiomes as a Paradigm for the Evolution of Terrestrialization

**DOI:** 10.3389/fmicb.2021.770245

**Published:** 2021-10-15

**Authors:** Matthew Wale, Daniele Daffonchio, Marco Fusi, Ramona Marasco, Elisa Garuglieri, Karen Diele

**Affiliations:** ^1^School of Applied Sciences, Edinburgh Napier University, Edinburgh, United Kingdom; ^2^Red Sea Research Center, King Abdullah University of Science and Technology, Thuwal, Saudi Arabia

**Keywords:** terrestrial crabs, symbiotic microbiota, hologenome theory, microbiome transmission, larval development

## Introduction

The transition from an aquatic to a terrestrial lifestyle has evolved multiple times, and in numerous different phyla, in earth's history. In many crab species, this process is still underway (Bliss and Mantel, 1968), providing a unique opportunity to study the evolution of terrestrialization, as well as the role of associated microbiomes during this process (Cannicci et al., 2020). Recently, Cannicci et al. (2020) reported on the potential importance of microbiomes in the transition of crabs, formally wholly aquatic species, to life, fully or in part, in terrestrial environments. The authors argue that symbiotic bacteria, such as those of gill and gut microbiomes, may play a key role in easing this transition, by helping crabs to overcome physiological and morphological challenges associated with conquering the terrestrial environment, such as impaired respiration and osmotic regulation, and a new, often primary plant-based low nitrogen diet. Here we focus on the microbiomes of crab larvae and their potential role for the evolution of terrestrialization.

Crabs that are transitioning to life on land fall into two broad categories: terrestrial species that spend their whole adult life (except for larval release) on land independent of tidal inundation or freshwater bodies, and semi-terrestrial species that spend their adult life on land but are dependent on tidal inundation or freshwater (Burggren and McMahon, 1988; Anger, 1995). Many marine organisms form symbiotic relationships with microorganisms to aid life in extreme environments (Sogin et al., 2020). In line with the hologenome theory, this suggests that host-microbe interactions play an important role in an organism's evolution, where the genes of both the host and its microbes co-evolve in the collective “holobiont” (Zilber-Rosenberg and Rosenberg, 2008), potentially allowing the colonization of formerly hostile environments (Bang et al., 2018). Microbial symbionts, as individual species or in mixed-species assemblages, are present in many crustaceans, such as the marine isopod *Idotea balthica* (diet-specific gut microbiomes, Mattila et al., 2014), the intertidal brachyuran crab *Eriocheir sinensis* (gill and gut microbiomes, Zhang et al., 2016), and the freshwater signal crayfish *Pacifastacus leniusculus* (intestinal bacteria, Hernández-Pérez et al., 2021). Given that microbial assemblages are often specific to certain organs of their hosts (Chomicki et al., 2020), symbioses have likely evolved in support of a specific function.

The microbial assemblages associated with the guts of semi-terrestrial crabs have been proposed to aid in the adaptation of a low nitrogen, herbivorous diet during terrestrialization (Bui and Lee, 2015), like microbial assemblages of other aquatic invertebrates, e.g., isopods, where they enable the digestion of cellulose (Zimmer et al., 2002; O'Connor et al., 2014). The bacteria specifically associated with crab gills (Zhang et al., 2016, 2017) may facilitate ammonia excretion (Weihrauch et al., 2004), utilize gaseous CO_2_ (Morris, 2001), and buffer exposure to oxygen, which occurs at a concentration 30 times higher (Hsia et al., 2013) in the terrestrial compared to the marine environment where the host organism evolved. The microbiomes of both gut and gills could therefore provide terrestrial and semi-terrestrial crabs (here collectively called semi-/terrestrial) with means to cope with life in marine as well as in terrestrial environments. Whilst the presence of microbiomes and their role in buffering the stresses imposed on crabs by terrestrialization is beginning to be discussed (Bui and Lee, 2015; Cannicci et al., 2020), there are many unknowns. For example, the mode of bacterial acquisition, bacterial diversity, topological association, and the precise functions of their organ-specific microbial assemblages are still poorly understood, both for adult semi-/terrestrial crabs and their early life stages.

Most semi-/terrestrial crab species, like their aquatic counterparts, have a biphasic life cycle including fully aquatic larvae, via which they transition to semi-/terrestrial juvenile/adult life (Anger, 1995; Hartnoll et al., 2014). Understanding microbial colonization of the larvae would likely provide critical insights into how these crabs have been able to move from water to land, and whether the bacteria themselves facilitate this transition.

## Larvae, the Vector Between Water and Land, and Their Potential Microbiome Acquisition Pathways

Most semi-/terrestrial crab species are broadcast spawners with pelagic larvae that develop for 3–6 weeks in the marine environment (Bliss and Mantel, 1968; Anger, 1995), undergoing multiple zoeal stages and one megalopal stage, mirroring the life history characteristics of fully aquatic crabs. In some semi-/terrestrial species, larval development is abbreviated (Anger, 1995; González-Gordillo et al., 2010; Vogt, 2013) and/or maternal brood care increased. For example, the larvae of the fiddler crab *Uca subcylindrica*, a species adapted to semiarid habitats, develop inside non-permanent water bodies to megalopae within as little as 2.5 days (Rabalais and Cameron, 1983). The Jamaican freshwater bromeliad crab *Metopaulias depressus* also undergoes abbreviated development; its larvae are released into phytothelmes and are actively guarded by their mothers (Diesel, 1989; Diesel and Schuh, 1993). Semi-/terrestrial crabs with larvae that (still) need to develop in the sea act as vectors at the marine-terrestrial interface. Some species travel considerable distances over land from their inland habitats to the coast, e.g., the inland forest-dwelling Christmas Island crab *Gecarcoidea natalis* travels up to 4 km (Adamczewska and Morris, 2001) to release its larvae into the sea. In the sea, the offspring of semi-/terrestrial crabs are preyed upon by aquatic predators and consume plankton to acquire biomass that is brought back to the land when the megalopae settle and metamorphose into the benthic juvenile stage. Hence, semi-/terrestrial crab larvae are couplers of nutrients, and likely microbiomes, between aquatic and terrestrial ecosystems, with each successive generation having to make the transition from a life in water to a life on land. If, as recently suggested (Cannicci et al., 2020), symbiotic microbiomes facilitate the evolution from an aquatic to a terrestrial life, then these larval stages provide a unique opportunity to explore this transition firsthand.

Bacterial colonization of marine invertebrate larvae has been evidenced as an essential step in their adaptation to specific, often extreme, environments. For example, once settled, the larvae and juveniles of the gutless giant tube worm *Riftia* spp. are colonized by sulfur-oxidizing bacteria (Nussbaumer et al., 2006). Their acquisition promotes developmental changes in the juvenile worm, including the growth of specific tissues for the bacteria to colonize. The endosymbiotic bacteria within these tissues facilitate sulfur-oxidation (Minic and Hervé, 2004), allowing the worms to survive in hydrothermal vent ecosystems. In some species, such as the sponge *Amphimedon queenslandica*, settlement and metamorphosis to benthic juvenile stage are facilitated by the larvae's microbiota (Song et al., 2021). These vertically acquired symbiotic bacteria produce arginine, the substrate needed for nitric oxide synthesis and the signal that regulates settlement and metamorphosis in many invertebrate species. Bacterial colonization plays a key role in the larvae of the intertidal barnacle *Semibalanus balanoides*, which are colonized during the settlement phase of the cyprid stage. The cypris' microbiome composition changes upon settlement, with potential implications for growth and survival during the barnacle's benthic life stage (Aldred and Nelson, 2019). In other marine crustacean species embryos and larvae are colonized by specific symbiotic bacteria that increase protection from pathogenic fungi and bacteria (Gil-Turnes et al., 1989—*Palaemon macrodactylus*; Gil-Turnes and Fenical, 1992—*Homarus americanus*), and in Bryozoa bacteria reduce larval predation risk through the production of cytotoxins (Lopanik et al., 2004—*Bugula neritina*).

While microbiomes have not been found in all animal species studied (including crustacean larvae) (Hammer et al., 2019; Martin et al., 2020), there is evidence that when present in marine invertebrate larvae, symbiotic relationships are often formed. It is important to comprehend at which developmental stage these microbes are acquired if we are to understand whether and how these relationships aid the transition between life stages and contribute to the evolution toward terrestrialization.

**Figure 1:**
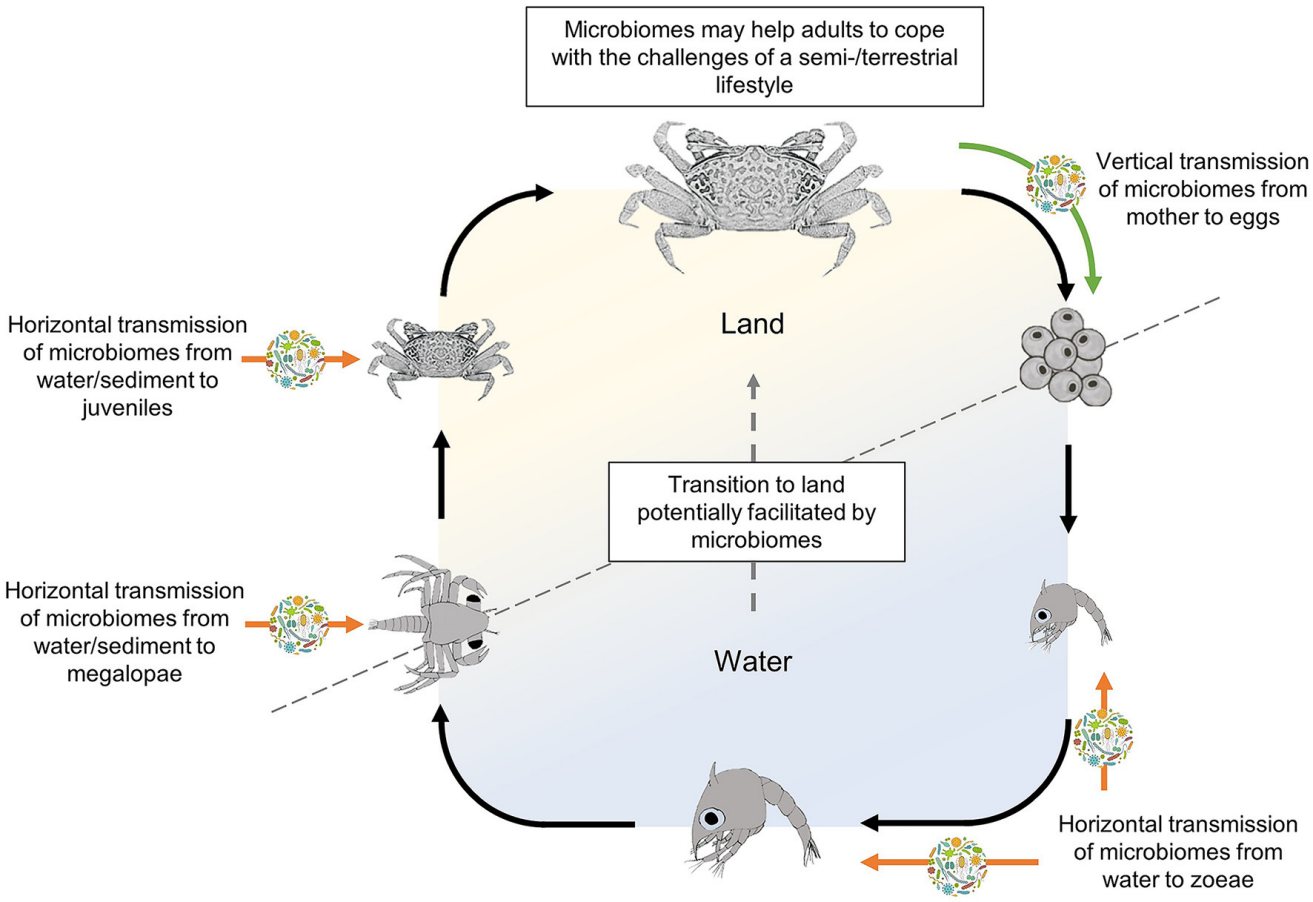
Life cycle of a semi-terrestrial crab (here represented as a female fiddler crab; subfamily Gelasiminae, taken as a model species; adapted after Peer et al., 2015) indicating the potential time points of vertical and horizontal acquisition of symbiotic microbiomes that may facilitate the transition from a life in water to one on land. Green and orange arrows indicate potential vertical and horizontal transmission routes of symbiotic microorganisms, respectively. Black arrows indicate the model life cycle from egg, through larvae, to adult crab.

Larval microbiomes of semi-/terrestrial crabs may be obtained horizontally from the surrounding environment, or vertically, through their parents (Figure 1). Horizontal bacterial transmission can occur either during the pelagic stages from the surrounding water (Lopanik et al., 2004; Hadfield, 2021) or upon first settlement (Nussbaumer et al., 2006; Aldred and Nelson, 2019) e.g., at the megalopa stage. Megalopae of many semi-/terrestrial crabs spend considerable time on the sediment surface, ahead of their metamorphosis into benthic juveniles (Anger, 2001; Hamasaki et al., 2015) and settlement is often driven by specific environmental and conspecific cues present in the sediment or the water layer above (Diele and Simith, 2007; Simith et al., 2013), possibly including substances emitted by bacterial biofilms (Simith et al., 2017). The period that the megalopae spend on the sediment before metamorphosing to the first juvenile crab stage may therefore provide an ideal time for the acquisition of bacteria from this substrate (Lim et al., 2019). Evidence of sediment bacteria transfer has been shown in adult fiddler crabs, *Uca panacea*, where the crabs' microbiome is populated by bacteria from their burrows (carapace microbiome) and the sediment surface (carapace and gut microbiome) (Cuellar-Gempeler and Leibold, 2019).

Alternatively, vertical transmission of a larva's microbiome is generally through the mother, either from her microbiota or that received from the father during mating (Damiani et al., 2008). Given the conservative broadcast spawning and pelagic larval stages of most semi-/terrestrial crabs, vertical transmission would have to occur through symbiont association with adult gametes (Russell et al., 2018) or through an association between the microbes and the females' ovaries. Egg associated microbiomes that act as the vertical transmission point for symbionts (Nyholm, 2020) have been evidenced for the deep-sea yeti crab, *Kiwa puravida* (Goffredi et al., 2014), and the hydrothermal vent shrimp, *Rimicaris exoculata* (Methou et al., 2019). In semi-/terrestrial crabs, microbes that are transmitted through the mother's eggs would colonize their target organ upon hatching or, if such organ has not yet developed at this developmental stage (e.g., gills), persist internally or on the larvae's carapace until formed. If vertical transmission is occurring in semi-/terrestrial crabs, it would indicate a complex symbiotic relationship where the crabs' entire life cycles are intertwined with their microbiome.

Given the large number of crab species that have conquered the land but retained pelagic larvae that develop in the oceans, we advocate the importance of integrating the early life history stages into future studies on microbiomes as a paradigm for the evolution of terrestrialization in crabs. Identifying whether, when, and under which acquisition pathway microbiome transmission is occurring in the early life history of species with biphasic life cycles has the potential to significantly enhance our understanding of the contribution of animal-microbiome interactions in the crabs' transition from a life in water onto land.

## Author Contributions

MW and KD conceived the idea for the paper. MW led the manuscript drafting, with input from all co-authors.

## Funding

This work received funding from King Abdullah University of Science and Technology Competitive Research Grant Program 2018—OSR-CRG 2018-3739.

## Conflict of Interest

The authors declare that the research was conducted in the absence of any commercial or financial relationships that could be construed as a potential conflict of interest.

## Publisher's Note

All claims expressed in this article are solely those of the authors and do not necessarily represent those of their affiliated organizations, or those of the publisher, the editors and the reviewers. Any product that may be evaluated in this article, or claim that may be made by its manufacturer, is not guaranteed or endorsed by the publisher.
